# Supplementary material for: A comparative genomic study of a hydrocarbon-degrading marine bacterial consortium
Source: PLoS One. 2024 Aug 8;19(8):e0303363. doi: 10.1371/journal.pone.0303363 (PMC11309472; doi:10.1371/journal.pone.0303363)
Supplement: S1 Fig — The functional prediction was made using the HADEG database and the RAST-tk annotation. (PDF) [file pone.0303363.s001.pdf]

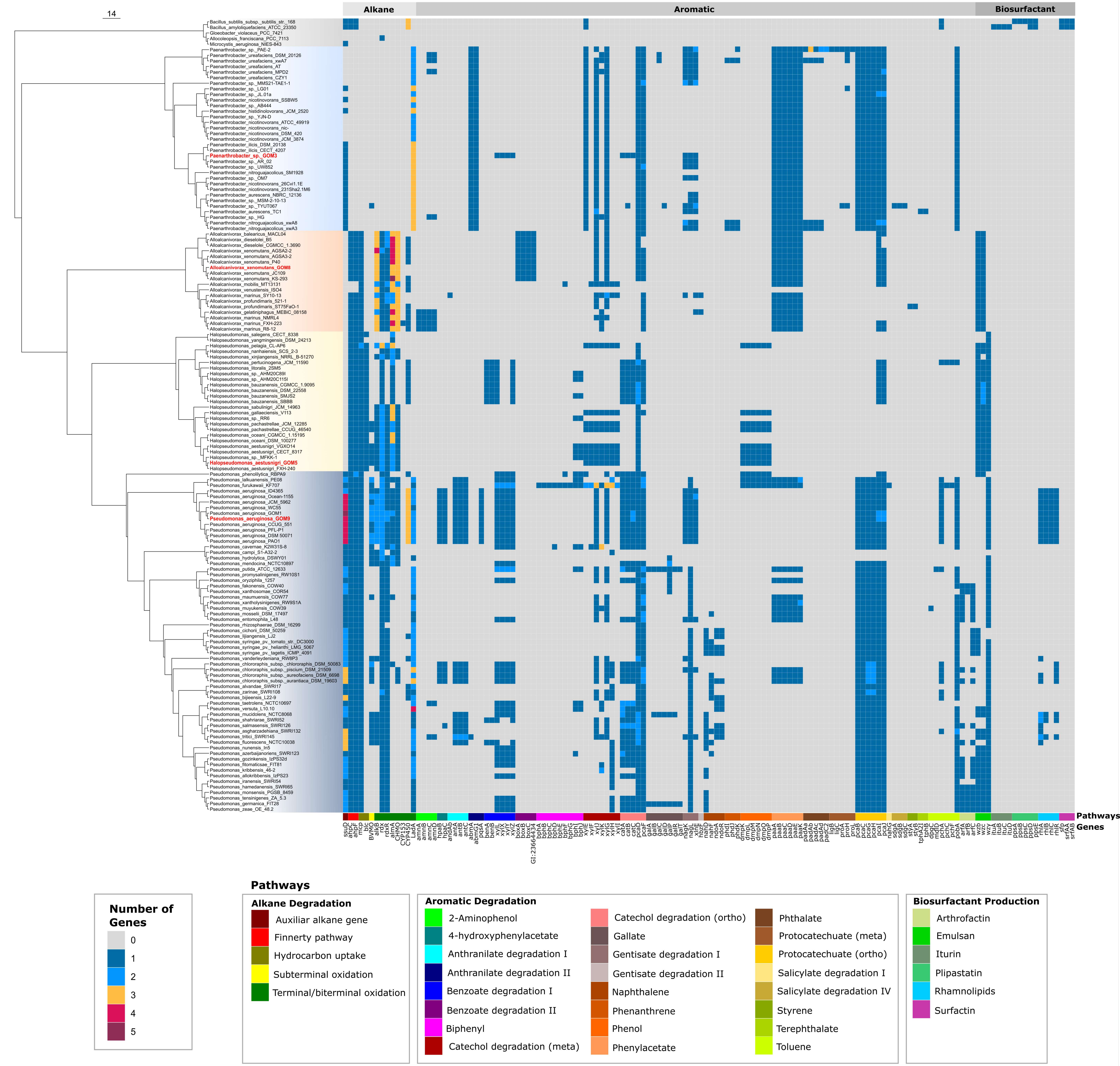

14
Alkane
Aromatic
Biosurfactant
Pathways
Genes
Number of Genes
0
1
2
3
4
5
Pathways
Alkane Degradation
Auxiliar alkane gene
Finnerty pathway
Hydrocarbon uptake
Subterminal oxidation
Terminal/biterminal oxidation
Aromatic Degradation
2-Aminophenol
4-hydroxyphenylacetate
Anthranilate degradation I
Anthranilate degradation II
Benzoate degradation I
Benzoate degradation II
Biphenyl
Catechol degradation (meta)
Catechol degradation (ortho)
Gallate
Gentisate degradation I
Gentisate degradation II
Naphthalene
Phenanthrene
Phenol
Phenylacetate
Phthalate
Protocatechuate (meta)
Protocatechuate (ortho)
Salicylate degradation I
Salicylate degradation IV
Styrene
Terephthalate
Toluene
Biosurfactant Production
Arthrofactin
Emulsan
Iturin
Plipastatin
Rhamnolipids
Surfactin
