# Supplementary material for: A comparative genomic study of a hydrocarbon-degrading marine bacterial consortium
Source: PLoS One. 2024 Aug 8;19(8):e0303363. doi: 10.1371/journal.pone.0303363 (PMC11309472; doi:10.1371/journal.pone.0303363)
Supplement: Attachment — Submitted filename: Comments Manuscript Number PONE D 23 38809.docx [file pone.0303363.s008.docx]

**Manuscript Number: PONE-D-23-38809**

**Title: A comparative genomic study of a hydrocarbon-degrading marine bacterial consortium**

**Comments**

1. The manuscript is well written; however, the author needs to address following comments
2. Improve the keywords, that should be reflected the nature of the work
3. Introduction part needs enhancement.
4. What are the potential sources of contamination, and how was this contamination assessed?
5. Check the typological error and language quality.
6. What was the specific methodology used to isolate the bacterial strains or give any references for isolation.
7. Rewrite the Consortium strains and nucleic acid extraction
8. How to conform the biosurfactant production of this bacterial strain (any specific analysis).
9. The images are not clear give high-DPI quality images.
10. Check the References are placed correctly.
