# Supplementary material for: A comparative genomic study of a hydrocarbon-degrading marine bacterial consortium
Source: PLoS One. 2024 Aug 8;19(8):e0303363. doi: 10.1371/journal.pone.0303363 (PMC11309472; doi:10.1371/journal.pone.0303363)
Supplement: Attachment — Submitted filename: Response to reviewers.docx [file pone.0303363.s009.docx]

**RESPONSE TO REVIEWERS**

Please find below the responses to the comments from the Reviewers:

**Reviewer #1**

In this manuscript, a study on a bacterial community isolated from a hydrocarbon contaminated marine site was conducted. The genome of four isolates was investigated in detail and a plethora of information was obtained, focusing on identification of genes encoding for proteins involved in hydocarbons degradation, allowing to shed light on the role isolated strains may have in oil hydrocarbons degradation and giving a base for possible bioremediation uses.

This study offers an interesting view of the role different members of a microbial consortium may perform, thus conducting to an efficient degradation of an organic complex mixture. Oil hydrocarbons with their aliphatic and aromatic components, their hydrophobic nature, their scarce bioavailability, can be used as carbon and energy source by heterotrophic bacteria.

The manuscript is well written and can give insights for future research.

**Line 25: ‘… 138 bacterial species …’ change to ‘… 138 bacterial stains …’**

Thank you for your observation. L25, now **L26**, has been modified by changing "species" to "strains."

Biosurfactants allow better bioavailability of hydrophibic substrata as hydrocarbons, in the meantime, adhesion of bacterial cells to hydrocarbons can represent an important aspect favoring bacterial degradation of hydrophibic substrata. Investigation in this context could add information on the efficiency of these isolates in degrading hydrocarbons in bioremediation processes.

**Reviewer #2**

1. The manuscript is well written; however, the author needs to address the following comments

**2. Improve the keywords, that should be reflected the nature of the work**

Thank you for the comment. The new keywords in **L38-39** are “marine bacterial consortium, hydrocarbon-degradation genes, functional diversity, comparative genomics, pangenome analysis”.

**3. Introduction part needs enhancement.**

The introduction was improved emphasizing the environmental impact of oil spills (**L41-43**). It also tightens the connection between HC pollution and bioremediation, focusing on the role of bacteria as a natural solution (**L44-48**). Additionally, complex sentences have been rephrased and redundant wording removed, improving overall readability (**L60-80**).

**4. What are the potential sources of contamination, and how was this contamination assessed?**

Regarding the first question, the new Introduction highlights in **L41:** "Crude oil spills, a consequence of anthropogenic activities in the ocean" acknowledging the human role in these environmental disasters.

For the second question, oil spill contamination can be assessed through different methods like visual observation, chemical analysis of water and sediment samples, and remote sensing [(NOAA 2020; Mauseth and Parker 2011)](https://paperpile.com/c/4PtWbN/AZwv+5ZOg). Our present research focuses on the bioremediation stage that follows the initial assessment. By understanding the genetic content of HC-degrading bacteria, we can develop more effective strategies to utilize these natural clean-up crews for future spills.

References

[Mauseth, Gary S., and Heather Parker. 2011. “Natural Resource Damage Assessment.” In *Oil Spill Science and Technology*, 1067–82. Elsevier.](http://paperpile.com/b/4PtWbN/5ZOg)

[NOAA. 2020. “Oil Spills.” August 1, 2020.](http://paperpile.com/b/4PtWbN/AZwv) <https://www.noaa.gov/education/resource-collections/ocean-coasts/oil-spills>[.](http://paperpile.com/b/4PtWbN/AZwv)

**5. Check the typological error and language quality.**

Thank you for your observation, the text was revised again, and we believe the errors were corrected.

**6. What was the specific methodology used to isolate the bacterial strains or give any references for isolation.**

Thank you for your question regarding the methodology used to isolate the bacterial strains. We apologize for not including this information in the previous draft. We have added a section under "Consortium strains and nucleic acid extraction" detailing the isolation process (**L84-88**). Briefly, the strains were isolated from the Gulf of Mexico using enrichment culture techniques followed by purification through streaking [reference the isolation method in reference 15]. We hope this clarifies our methodology.

7. Rewrite the Consortium strains and nucleic acid extraction

Done as we described in the previous question.

8. How to conform the biosurfactant production of this bacterial strain (any specific analysis).

While our study focused on predicting the metabolic potential for hydrocarbon degradation and biosurfactant production, confirming biosurfactant production through specific analyses (e.g., droplet collapse assay, and surface tension measurement) would be a valuable addition to future research but was not part of the main scope of this work. We added this suggestion in **L485-488**.

9. The images are not clear give high-DPI quality images.

Done. The new version has all images in high quality according to the PACE tool by PLoS.

10. Check the References are placed correctly.

The text has been reviewed, and we believe the References are placed correctly.
